# The Emerging Role of Lamin C as an Important *LMNA* Isoform in Mechanophenotype

**DOI:** 10.3389/fcell.2018.00151

**Published:** 2018-11-02

**Authors:** Rafael D. González-Cruz, Kris N. Dahl, Eric M. Darling

**Affiliations:** ^1^Center for Biomedical Engineering, Brown University, Providence, RI, United States; ^2^Department of Chemical Engineering, Department of Biomedical Engineering, Carnegie Mellon University, Pittsburgh, PA, United States; ^3^Department of Molecular Pharmacology, Physiology and Biotechnology, School of Engineering, Department of Orthopaedics, Brown University, Providence, RI, United States

**Keywords:** nuclear lamins, cell stiffness, mechanical properties, biomarkers, ratiometric isoform expression, laminopathies

## Abstract

Lamin A and lamin C isoforms of the gene *LMNA* are major structural and mechanotransductive components of the nuclear lamina. Previous reports have proposed lamin A as the isoform with the most dominant contributions to cellular mechanophenotype. Recently, expression of lamin C has also been shown to strongly correlate to cellular elastic and viscoelastic properties. Nevertheless, *LMNA* isoforms exist as part of a network that collectively provides structural integrity to the nucleus and their expression is ultimately regulated in a cell-specific manner. Thus, they have importance in mechanotransduction and structural integrity of the nucleus as well as potential candidates for biomarkers of whole-cell mechanophenotype. Therefore, a fuller discussion of lamin isoforms as mechanophenotypic biomarkers should compare both individual and ratiometric isoform contributions toward whole-cell mechanophenotype across different cell types. In this perspective, we discuss the distinctions between the mechanophenotypic correlations of individual and ratiometric lamins A:B1, C:B1, (A + C):B1, and C:A across cells from different lineages, demonstrating that the collective contribution of ratiometric lamin (A + C):B1 isoforms exhibited the strongest correlation to whole-cell stiffness. Additionally, we highlight the potential roles of lamin isoform ratios as indicators of mechanophenotypic change in differentiation and disease to demonstrate that the contributions of individual and collective lamin isoforms can occur as both static and dynamic biomarkers of mechanophenotype.

## Introduction

Nuclear lamina proteins are type V intermediate filament proteins that exhibit important nuclear roles by contributing to structural integrity and regulating transcriptional activities (Dechat et al., 2010). A-type lamins are expressed from the gene *LMNA* and include primarily lamin A and lamin C, although other minority isoforms and splice variants occur naturally as well (Worman, 2012; DeBoy et al., 2017). Also, most commercially available antibodies recognize both lamins A and C, so most cellular immunolabeling does not distinguish the isoforms in labeled cells. Conversely, B-type lamins, such as lamin B1 and lamin B2, are differentially expressed by *LMNB1* and *LMNB2* and can be readily imaged together or separately. These proteins include lamin isoforms A, B1, B2, and C and are expressed at variable levels in all mammalian cells (Lin and Worman, 1993, 1995). Together, these isoforms interact with several nuclear membrane proteins to form the nuclear lamina, although the A-type and B-type proteins form independent filaments, and filament networks are spatially segregated within the nuclear lamina (Shimi et al., 2008). From a structural standpoint, lamins are connected to a network of intermembrane proteins that form the linker of the nucleus to cytoskeleton (LINC) protein complex, which is itself connected to the actomyosin cytoskeleton (Lombardi et al., 2011). Because of these connections, lamin proteins not only relay physical cues from the external microenvironment to the nucleus to induce physical chromatin rearrangement and influence gene expression but also associate with perinuclear actin-LINC supramolecular complexes to prevent nuclear deformation upon exposure to these mechanical cues (Dahl et al., 2008; Osmanagic-Myers et al., 2015; Alam et al., 2016; Kim et al., 2017).

## Lamins and Mechanophenotype

Previous research has identified that lamin proteins A and C are important for imparting the nucleus with its stiffness, and their expression has been reported to scale with tissue stiffness (Swift et al., 2013). It has also been shown that *LMNA* is upregulated when cells are seeded on stiff substrates as well as when stem cells are induced to differentiate into mechanically less compliant cell types (Swift et al., 2013; Swift and Discher, 2014). *LMNA* gene mutations that prevent the expression or synthesis of mature lamin A filaments result in defective mechanotransduction and enhanced nuclear fragility that arises from severing actin/LINC-lamin A/C interactions (Lammerding et al., 2004; Kim et al., 2017). Mutations known to cause human disease exist all along the *LMNA* gene, collectively known as laminopathies; over 100 different mutations lead to over a dozen different diseases. Some of these diseases are mechano-weakening and some are mechano-stiffening disorders, and some mutations have no apparent mechanophenotype (Dahl et al., 2008). Interestingly, the creation of a transgenic mouse known as a lamin C-only mouse allowed for consideration of expression of the lamin C isoform but not the lamin A isoform of *lmna*. Aside from mild nuclear fragility, this mouse showed none of the characteristic defects associated with muscular dystrophy observed in the full knockout of *lmna*.

Motivated by the difference in lamin A and lamin C in transgenic mice, studies that have looked into individual contributions have found that both lamin A and C are important for mechanophenotype, although with certain discrepancies between their findings (Fong et al., 2006; Lammerding et al., 2006; Swift et al., 2013; Gonzalez-Cruz et al., 2018). Some of the studies suggest lamin A is the most dominant mechanophenotypic contributor, as lamin A protein expression has been demonstrated to scale strongly with tissue microelasticity (Swift et al., 2013). Meanwhile, more recent studies present evidence for lamin C as the strongest correlate to whole-cell mechanophenotype and most sensitive molecule for mechanophenotypic changes (Cho et al., 2018; Gonzalez-Cruz et al., 2018). It is possible that discrepancies between studies could result from differences in protein extraction protocols (Janes, 2015; Gonzalez-Cruz et al., 2018). Regardless of these discrepancies, the most important common finding from these studies is that the expression of lamins is dependent on cell/tissue lineage, not only in static scenarios such as biomarker characterization but also on dynamic situations like stem cell differentiation and disease progression (Swift et al., 2013; Gonzalez-Cruz et al., 2018).

While individual lamin A and C, but not lamin B1 and B2, isoform expressions can serve as a proxy for cellular mechanical properties, it is important to remember that these isoforms exist as part of a composite meshwork that collectively contributes to mechanophenotype. Even more important, while lamin A and C have been shown by different groups to correlate to mechanophenotype, the expression of lamin A and C isoforms is not equal across all cell types. Because of cell-dependent expression of these isoforms and their aggregate contributions to mechanotransduction, the ratiometric expression of lamin isoforms should also be considered, especially when these ratios could change, evenly or not, during cell differentiation and disease progression, thus indicating a major phenotypic shift (Bermeo et al., 2015; Aljada et al., 2016). It is plausible for cells undergoing such changes in lamin expression to exhibit an isoform shift in their individual and collective lamin ratios that is concomitant with the change to a newly acquired phenotype. This is especially important if the expression of one isoform is favored over another based on either lineage-specific or pathological mechanophenotype.

## Lamin C is a Unique Filament that Assembles Last

Consideration of lamin C as an important mechanical element within the cell and nuclear lamina requires a deeper understanding of its structure and integration into the lamina network. Lamin C is the only of the lamins not to be post-translationally modified with a farnesylated tail domain. Lamins B1 and B2 maintain their tails, and prelamin A undergoes a cleavage once it is inside the nucleus to lose the modified tail domain. Because lamin C lacks this farnesylated tail domain, its expression is unaffected by certain mutations that occur in genes coding for the tail domain or affecting farnesylases, which is a feature of certain lamin A-specific diseases (Dechat et al., 2008). Also, the filaments formed from each protein are homofilaments: lamin C only associates with lamin C, lamin A and C do not form heterodimeric complexes (Pugh et al., 1997).

**FIGURE 1:**
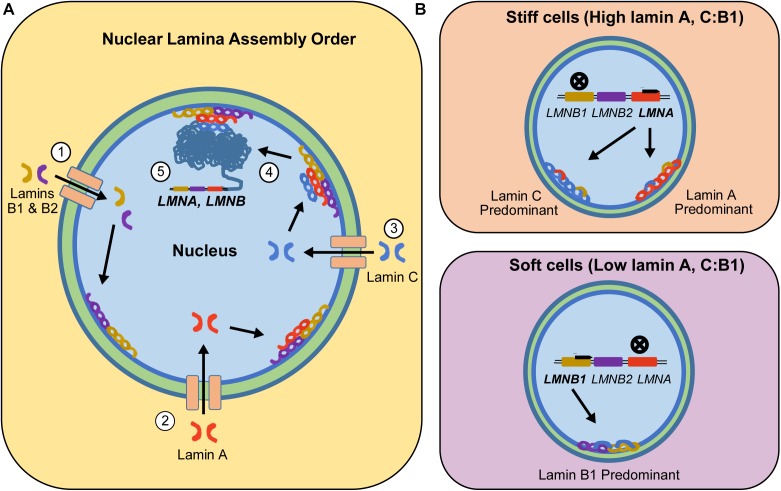
Step-wise incorporation of lamin isoforms into the nuclear lamina and resulting isoform expression based on cells’ inherent mechanophenotype. **(A)** B-type lamins enter the nucleus and form the first layer of the nuclear lamina (Step 1). The incorporation of B-type lamins into the nuclear lamina allows them to recruit internalized lamin A isoforms into the nuclear lamina (Step 2). Once lamin A is part of the nuclear lamina, internalized lamin C proteins can attach to the nuclear lamina meshwork (Step 3). All these recruited lamin proteins generate intracellular tension that is relayed into the chromatin network (Step 4), which in part regulates expression of lamins to match the characteristic levels of a given mechanophenotype (Step 5). **(B)** This intracellular tension-led regulation will upregulate LMNA genes in stiff cells, resulting in cells expressing higher levels of lamin A or C. However, in soft cells, intracellular tension is low, resulting in downregulation of LMNA and upregulation of LMNB1. During these processes, lamin isoforms assemble as homodimers and inter-lamin interactions with the nuclear lamina are facilitated by lamin-binding and other accessory proteins.

Lamins are incorporated into the lamina meshwork at different times during nuclear envelope assembly (Figure 1; Vaughan et al., 2001). Specifically, lamin B1 and B2 are the first lamins recruited to the nuclear meshwork following nuclear envelope breakdown and reformation in mitosis, followed by lamin A and then lamin C (Shimi et al., 2015). The presence of lamin A and B1 in the lamina is required for successful recruitment of lamin C (Pugh et al., 1997). However, the presence of one isoform affects the expression of the other. Previous studies using 3T3 fibroblast cell lines indicated that once lamin C is incorporated into the lamina meshwork, lamin A mRNA synthesis is reduced (Pugh et al., 1997). One possible hypothesis for this result is that, as lamin C is incorporated into the nuclear lamina, it alters the intranuclear tension experienced by the nucleus. Based on previous literature, the nuclei can readjust its lamin A concentration to rebalance the intracellular tension to levels that are appropriate for a given phenotype (Buxboim et al., 2014; Dingal and Discher, 2014). Generally, although all of the lamins do not interact directly there is thought to be some regulation of expression by overexpression of other lamins either by mechanics, space constraints, or other regulatory pathways.

## Individual and Aggregate Contributions of Lamins to Mechanophenotype

At first glance, the structural roles of lamins in maintaining nuclear integrity and mechanotransduction strongly suggest that their expression should be intrinsically tied to cellular mechanophenotype. This hypothesis has proven to be true in previous studies for 2 of the 4 lamins: A and C (Lammerding et al., 2004, 2006; Lammerding and Lee, 2005). Additionally, these lamins have been shown to have roles in establishing nuclear mechanotransduction and stiffness (Lammerding et al., 2006; Swift et al., 2013). However, when each isoform was examined for their individual contributions to mechanophenotype, different studies have reported unequal contributions to mechanophenotype. In some studies, individual lamin A and total lamin A/C expression has being proposed as the most important contributor to mechanophenotype. We have found that individual lamin C to correlate better with mechanical properties than lamin A (Gonzalez-Cruz et al., 2018). On the other hand, previous studies, including our own, have shown that lamin B1 and B2 isoforms have no significant influence in cellular mechanophenotype (Lammerding et al., 2006; Swift et al., 2013; Gonzalez-Cruz et al., 2018).

**FIGURE 2:**
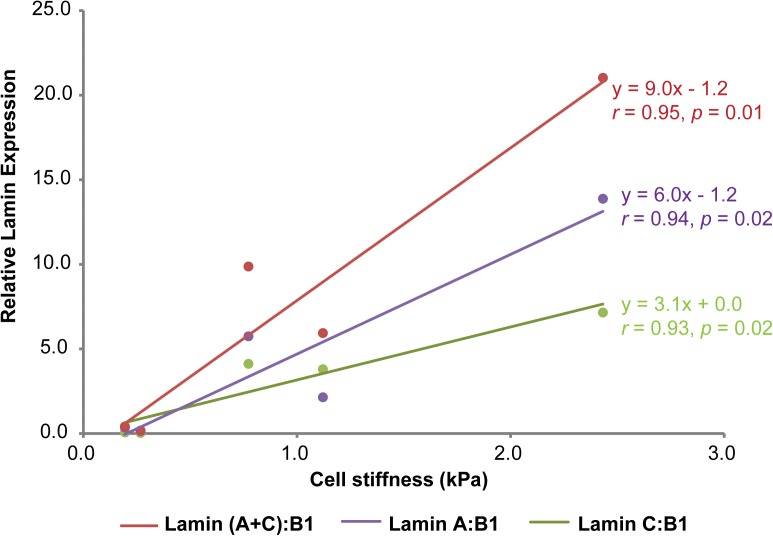
Correlations between lamin A:B1, C:B1, (A + C):B1, and C:A and cell stiffness. Relative protein expression for lamins A, B1, and C was determined via western blot and densitometry analysis from protein lysates (10 μg) extracted from osteoblastic (MG-63), fibroblastic (NHF), ovarian (KGN), renal (HEK-293T) and neuronal (SH-SY5Y) cell lines using a urea-sodium dodecyl lysis buffer, as described elsewhere (Gonzalez-Cruz et al., 2018). Individual protein expressions were normalized to beta tubulin loading control expression prior to isoform ratiometric analyses. Mechanophenotype was determined by measuring the elastic modulus of MG-63, NHF, KGN, HEK-293T, and SH-SY5Y cells via atomic force microscopy single-cell indentation. Lamin B1, a mechanically unresponsive lamin isoform that is important for nuclear lamina assembly was used as a normalizing factor to re-assess the contributions of lamin A and C to whole-cell mechanophenotype (Lammerding et al., 2006). Data points shown as arithmetic means. Correlation analyses between cell stiffness and ratiometric lamin expression were determined by calculating Pearson’s *r* coefficient for each set of comparisons. Statistical significance for the *r* coefficients was determined from Student’s *t* distribution probability function calculations.

While the individual lamin isoform-mechanophenotype correlations from our study suggest that lamin C is a better correlate of mechanophenotype, we also wanted to understand if these findings were also true at the lamin aggregate assembly and expression levels, since that is how these proteins would be found in living cells. To test this hypothesis, we explored the numerical interrelationships of lamin A, C, and A + C isoform expressions normalized to lamin B1 and whole-cell mechanophenotype. In our original study, protein expression level for each lamin was quantified using densitometry analysis of associated immunoblots (Gonzalez-Cruz et al., 2018). Average lamin A/B1/C levels could be plotted against average elastic moduli values for each of five different cell lines that span a range of whole-cell mechanophenotypes (Figure 2). After comparing correlations between lamin A:B1 and lamin C:B1 ratios toward mechanophenotype, we found that both lamin ratios correlate very strongly (and similarly) to mechanophenotype, with their combined expression, lamin (A + C):B1, correlating the strongest to whole-cell mechanophenotype. However, while both lamin A:B1 and C:B1 correlate strongly to mechanophenotype, their expression as a function of cell stiffness is variable across cells from different lineages (Figure 1). Lamin A:B1 ratios displayed a higher rate of change in their expression due to changes in cells stiffness than those reported for lamin C:B1 (*m_LA:LB1_* = 6.0 vs. *m_LC:LB1_* = 3.1). This result suggests that the lamin A isoform is more sensitive to mechanophenotypic changes than lamin C. Nevertheless, there is evidence to suggest this behavior holds for lamin A and C isoforms. In a previous study, we observed that disruption of the actin cytoskeleton using cytochalasin D resulted in a reduction in cell stiffness that was concomitant to 2.0- and 2.5-fold lower lamin C and A protein expression, respectively, in CytoD-stiff cells (Gonzalez-Cruz et al., 2018). Other studies have successfully demonstrated that lowering intracellular tension, via alteration of the matrix stiffness, drives the phosphorylation and degradation of both lamin A and C isoforms in a manner consistent with our observations in normal cells but not in cells with *LMNA* mutations, where the trend in mechanosensitivity is reversed (Buxboim et al., 2014; Cho et al., 2018). Altogether, these findings suggest that ratiometric analyses of isoforms are an advantageous way to analyze isoform expression without worrying about the differences in protein extraction buffers used.

## Lamin Isoform Ratio Switching: a Biomarker for Tracking Phenotypic Changes in Differentiation and Disease?

Since *LMNA* gene expression and nuclear stiffness can change drastically as stem cells differentiate, it has been hypothesized that changes in lamin isoform ratios could reveal aspects of lineage commitment, perhaps similarly to how cellular mechanical properties predict lineage differentiation potential in stem cell populations (Pajerowski et al., 2007; Gonzalez-Cruz et al., 2012). In the case of mesenchymal stem cells, *LMNA* is *upregulated* during osteogenesis but *downregulated* during adipogenesis (Swift et al., 2013; Bermeo et al., 2015). The upregulation of *LMNA* results in higher levels of lamin A and C in osteoblasts than in undifferentiated stem cells (Swift et al., 2013). Despite the overall increment of *LMNA* mRNA transcripts, the lamin A isoform is expressed at higher levels than its lamin C counterpart in differentiated, stiff osteoblasts. Conversely, soft cells types, like differentiated neurons, express significantly lower levels of lamin A than lamin C and B1 (Lee et al., 2016). Based on these reports, cells with different mechanophenotypes may experience dynamic changes in lamin assembly that could be indicative of differentiation fate. In the case of central nervous system (CNS) cells such as neurons and glia, it is possible that lamin A and C are expressed and incorporated into the nuclear lamina during the earlier stages of differentiation followed by significant downregulation of lamin A expression to exhibit a neurogenic mechanophenotype. This possibility is likely since the only cells that are known to continue expressing both lamin A and C at high levels in the brain under normal physiological conditions are endothelial and meningeal cells. Cells within the cerebellum and cerebral cortex that express lamin C, but not lamin A, have been observed to exhibit robust expression of astrocyte-specific glial fibrillary acidic protein (GFAP) and neuron-specific neuronal nuclei (NeuN) protein (Jung et al., 2012). Upregulation of lamin A and C in CNS cells has been observed in astrocytes from patients with Alexander disease, which is characterized by a gain-of-function mutation to the *GFAP* gene. While the *LMNA* gene itself is not mutated in this disease, brain tissues from mice models of Alexander disease exhibit higher lamin A and, specifically, higher lamin C expressions than normal brain tissues. These affected tissues expressing higher levels of lamin C were also found to be stiffer than those from wild-type mice (Wang et al., 2018). This finding would be in agreement with aforementioned connections between lamin A/C expression and stiffness (Swift et al., 2013; Gonzalez-Cruz et al., 2018). While these findings indicate that *LMNA* gene expression is upregulated in Alexander disease-mutated astrocytes, the fact that the lamin C isoform is expressed at a disproportionately higher level than lamin A suggests that the *LMNA* mRNA splicing in these GFAP-mutated cells favors lamin C expression over that of lamin A, even more than it already did in normal astrocytes (Jung et al., 2012; Wang et al., 2018). Altogether, this body of information suggests that lamin isoform ratios can shift as a result of changes in mechanophenotype. Additionally, since mechanophenotype can predict differentiation potential (Gonzalez-Cruz et al., 2012; Labriola and Darling, 2015), lamin isoform shifts could potentially serve as indicators of differentiation potential as well.

In the context of disease, laminopathies can result when mutations introduced into the *LMNA* gene prevent the correct synthesis of mature lamin A and C proteins (Ostlund et al., 2001). Since these mutations impair mechanotransduction, many of the laminopathic diseases affect tissues with load-bearing functionality like muscle and bone, in lamin A > C, but not others like the brain, in which lamin C > A (Young et al., 2012; Gonzalez-Cruz et al., 2018). In many of these laminopathies, such as Hutchison-Gilford progreria syndrome (HGPS) and dilated cardiomyopathy, mutations of the *LMNA* gene end up affecting one isoform more than another (Sylvius et al., 2008). In the case of HGPS, mutated cells exhibit accelerated aging and brittle nuclei because of a point mutation in exon 11 that prevents the synthesis of mature lamin A (De Sandre-Giovannoli et al., 2003). The resulting immature lamin A isoform, known as progerin, cannot be fully integrated into the lamina meshwork but accumulates and stiffens the nucleus. Previous studies suggest that this nuclear stiffening impaired mechanotransduction because the accumulated progerin molecules dampen the propagation of external forces into the interior of nuclei, thus making the nucleus less responsive to mechanical stimuli (Goldman et al., 2004; Booth et al., 2015). When these HGPS cells are compared to those undergoing normal aging, the mutated cells display higher levels of progerin than lamin C expression, suggesting that cells undergoing abnormal aging would exhibit a change in their lamin C:progerin ratios that favors the overexpression of the progerin over lamin C (Lee et al., 2016). This has being further demonstrated by recent work reporting that lamin C phosphorylates faster than progerin when intracellular tension is low or is lost in early-passage cells (Cho et al., 2018). Therefore, this isoform ratio switch could be an indicator of aging in cells and could be targeted to ameliorate some of the pathological effects of this disease. Specifically, if experimental designs such as insertion of microRNA miR-9, SRSF2 siRNA, or splice-switching exon 11 antisense oligonucleotides (ASO E-11) are implemented to increase the lamin C:progerin transcript ratio, pathological phenotypes associated with progeria and aging could be ameliorated by preventing the translation of faulty *LMNA* mRNA splice variants into progerin (Scaffidi and Misteli, 2005; Jung et al., 2012; Lee et al., 2016). Lamin C-specific mutations leading to disease can occur as well, although their effects on mechanophenotype remain unknown, although clinical cases reported so far suggest that the mutations do not affect lamin A:C mRNA splicing (Patni et al., 2017). Patients harboring these mutations can exhibit neuropathy and lipodystrophy, which are diseases that particularly affect cells that are not usually affected by widely reported laminopathies that result from mutations affecting lamin A synthesis and incorporation to the nuclear lamina meshwork (Ng and Kaye, 2013; Patni et al., 2017).

Other diseases like cancer can also occur when *LMNA* gene expression is dysregulated. One of the phenotypic traits of malignant cancer cells is their ability to metastasize. It has been proposed and demonstrated that nuclei from metastatic cells can change their lamin A and C expression levels to migrate from tissues to the bloodstream and vice versa into other tissues without experiencing irreversible damage and rupture (Harada et al., 2014). This hypothesis has been successfully tested through experiments in which ΔLA50, a mutated *LMNA* variant expressed in advanced aged and overexpressed in HGPS patients, was inserted in melanoma cells, resulting in nuclear stiffening but reduced metastatic potential of the cells (Ribeiro et al., 2014). In another study, SH-SY5Y neuroblastoma cells, which express lamin A and C at higher levels than healthy neurons, are shown to increase their metastatic potential if their *LMNA* gene expression and cell stiffness are reduced while gaining stem-like properties similar to tumor initiating cells (Maresca et al., 2012). Altogether, metastatic cells are sensitive to changes in *LMNA* gene expression, and since total lamin A + C expression is strongly correlated to mechanophenotype, the mechanophenotype of the cells is also sensitive to these changes. Yet, the picture becomes more complicated as recent studies suggest that lamin A and C isoforms might be expressed differently in various cancer types (Moss et al., 1999; Venables et al., 2001; Tilli et al., 2003; Willis et al., 2008; Aljada et al., 2016; Zuo et al., 2018). In breast, liver, and ovary cancers, the ratio of lamin C-derived mRNA splice variant to that of lamin A-derived mRNA splice variant is greater in metastatic cells than their healthy counterparts as *LMNA* mRNA splice variants coding for lamin C isoforms increase while lamin A isoforms decrease (Aljada et al., 2016). Therefore, isoform switching might be an important signature of disease onset and progression as well as a target for which therapeutic strategies can be designed to shift the aberrant lamin isoform ratio to approximate that of healthy cells.

## Conclusion

Lamin A and C isoforms show great promise as biomarkers of mechanophenotype when considering their expression as ratios of the entire lamin meshwork. Analyzing lamin A and C isoform ratios could provide end-point analysis metrics of whole-cell mechanophenotype in the context of lineage and/or microenvironment. On the other hand, monitoring isoform ratio shifts throughout time could illustrate dynamic changes in mechanophenotype that serve as biological signatures of disease onset or progression and lineage commitment of differentiating cells. Therefore, future experiments should consider the joint contributions of lamin isoforms to mechanophenotype as well as any instances of isoform switching that could be useful to predict phenotypic changes.

## Author Contributions

RG-C, KD, and ED contributed to this perspective, originating from work conducted by RG-C and ED. RG-C wrote the first draft of the manuscript, with KD and ED contributed to manuscript revisions. All authors approved the final version.

## Conflict of Interest Statement

The authors declare that the research was conducted in the absence of any commercial or financial relationships that could be construed as a potential conflict of interest.

## References

[B1] Alam S. G. Zhang Q. Prasad N. Li Y. Chamala S. Kuchibhotla R. (2016). The mammalian LINC complex regulates genome transcriptional responses to substrate rigidity. *Sci. Rep.* 6:38063. 10.1038/srep38063 27905489 PMC5131312

[B2] Aljada A. Doria J. Saleh A. M. Al-Matar S. H. Algabbani S. Shamsa H. B. (2016). Altered Lamin A/C splice variant expression as a possible diagnostic marker in breast cancer. *Cell. Oncol.* 39 161–174. 10.1007/s13402-015-0265-1 26732077 PMC13001873

[B3] Bermeo S. Vidal C. Zhou H. Duque G. (2015). Lamin A/C acts as an essential factor in mesenchymal stem cell differentiation through the regulation of the dynamics of the wnt/beta-catenin pathway. *J. Cell. Biochem.* 116 2344–2353. 10.1002/jcb.25185 25846419

[B4] Booth E. A. Spagnol S. T. Alcoser T. A. Dahl K. N. (2015). Nuclear stiffening and chromatin softening with progerin expression leads to an attenuated nuclear response to force. *Soft Matter* 11 6412–6418. 10.1039/c5sm00521c 26171741

[B5] Buxboim A. Swift J. Irianto J. Spinler K. R. Dingal P. C. Athirasala A. (2014). Matrix elasticity regulates lamin-A,C phosphorylation and turnover with feedback to actomyosin. *Curr. Biol.* 24 1909–1917. 10.1016/j.cub.2014.07.001 25127216 PMC4373646

[B6] Cho S. Abbas A. Irianto J. Ivanovska I. L. Xia Y. Tewari M. (2018). Progerin phosphorylation in interphase is lower and less mechanosensitive than lamin-A,C in iPS-derived mesenchymal stem cells. *Nucleus* 9 230–245. 10.1080/19491034.2018.1460185 29619860 PMC5973135

[B7] Dahl K. N. Ribeiro A. J. Lammerding J. (2008). Nuclear shape, mechanics, and mechanotransduction. *Circ. Res.* 102 1307–1318. 10.1161/CIRCRESAHA.108.173989 18535268 PMC2717705

[B8] De Sandre-Giovannoli A. Bernard R. Cau P. Navarro C. Amiel J. Boccaccio I. (2003). Lamin a truncation in Hutchinson-Gilford progeria. *Science* 300:2055. 10.1126/science.1084125 12702809

[B9] DeBoy E. Puttaraju M. Jailwala P. Kasoji M. Cam M. Misteli T. (2017). Identification of novel RNA isoforms of LMNA. *Nucleus* 8 573–582. 10.1080/19491034.2017.1348449 28857661 PMC5703264

[B10] Dechat T. Adam S. A. Taimen P. Shimi T. Goldman R. D. (2010). Nuclear lamins. *Cold Spring Harb. Perspect. Biol.* 2:a000547. 10.1101/cshperspect.a000547 20826548 PMC2964183

[B11] Dechat T. Pfleghaar K. Sengupta K. Shimi T. Shumaker D. K. Solimando L. (2008). Nuclear lamins: major factors in the structural organization and function of the nucleus and chromatin. *Genes Dev.* 22 832–853. 10.1101/gad.1652708 18381888 PMC2732390

[B12] Dingal P. C. Discher D. E. (2014). Systems mechanobiology: tension-inhibited protein turnover is sufficient to physically control gene circuits. *Biophys. J.* 107 2734–2743. 10.1016/j.bpj.2014.10.042 25468352 PMC4255197

[B13] Fong L. G. Ng J. K. Lammerding J. Vickers T. A. Meta M. Cote N. (2006). Prelamin A and lamin A appear to be dispensable in the nuclear lamina. *J. Clin. Invest.* 116 743–752. 10.1172/JCI27125 16511604 PMC1386109

[B14] Goldman R. D. Shumaker D. K. Erdos M. R. Eriksson M. Goldman A. E. Gordon L. B. (2004). Accumulation of mutant lamin A causes progressive changes in nuclear architecture in Hutchinson-Gilford progeria syndrome. *Proc. Natl. Acad. Sci. U.S.A.* 101 8963–8968. 10.1073/pnas.0402943101 15184648 PMC428455

[B15] Gonzalez-Cruz R. D. Fonseca V. C. Darling E. M. (2012). Cellular mechanical properties reflect the differentiation potential of adipose-derived mesenchymal stem cells. *Proc. Natl. Acad. Sci. U.S.A.* 109 E1523–E1529. 10.1073/pnas.1120349109 22615348 PMC3386052

[B16] Gonzalez-Cruz R. D. Sadick J. S. Fonseca V. C. Darling E. M. (2018). Nuclear lamin protein C is linked to lineage-specific, whole-cell mechanical properties. *Cell. Mol. Bioeng.* 11 131–142. 10.1007/s12195-018-0518-y 29755599 PMC5943047

[B17] Harada T. Swift J. Irianto J. Shin J. W. Spinler K. R. Athirasala A. (2014). Nuclear lamin stiffness is a barrier to 3D migration, but softness can limit survival. *J. Cell Biol.* 204 669–682. 10.1083/jcb.201308029 24567359 PMC3941057

[B18] Janes K. A. (2015). An analysis of critical factors for quantitative immunoblotting. *Sci. Signal.* 8:rs2 10.1126/scisignal.2005966 PMC4401487 25852189

[B19] Jung H. J. Coffinier C. Choe Y. Beigneux A. P. Davies B. S. Yang S. H. (2012). Regulation of prelamin A but not lamin C by miR-9, a brain-specific microRNA. *Proc. Natl. Acad. Sci. U.S.A.* 109 E423–E431. 10.1073/pnas.1111780109 22308344 PMC3289373

[B20] Kim J. K. Louhghalam A. Lee G. Schafer B. W. Wirtz D. Kim D. H. (2017). Nuclear lamin A/C harnesses the perinuclear apical actin cables to protect nuclear morphology. *Nat. Commun.* 8:2123. 10.1038/s41467-017-02217-5 29242553 PMC5730574

[B21] Labriola N. R. Darling E. M. (2015). Temporal heterogeneity in single-cell gene expression and mechanical properties during adipogenic differentiation. *J. Biomech.* 48 1058–1066. 10.1016/j.jbiomech.2015.01.033 25683518 PMC4380682

[B22] Lammerding J. Fong L. G. Ji J. Y. Reue K. Stewart C. L. Young S. G. (2006). Lamins A and C but not lamin B1 regulate nuclear mechanics. *J. Biol. Chem.* 281 25768–25780. 10.1074/jbc.M513511200 16825190

[B23] Lammerding J. Lee R. T. (2005). The nuclear membrane and mechanotransduction: impaired nuclear mechanics and mechanotransduction in lamin A/C deficient cells. *Novartis Found. Symp.* 264 264–273; discussion 273–268 10.1002/0470093765.ch18 15773759

[B24] Lammerding J. Schulze P. C. Takahashi T. Kozlov S. Sullivan T. Kamm R. D. (2004). Lamin A/C deficiency causes defective nuclear mechanics and mechanotransduction. *J. Clin. Invest.* 113 370–378. 10.1172/JCI200419670 14755334 PMC324542

[B25] Lee J. M. Nobumori C. Tu Y. Choi C. Yang S. H. Jung H. J. (2016). Modulation of LMNA splicing as a strategy to treat prelamin A diseases. *J. Clin. Invest.* 126 1592–1602. 10.1172/JCI85908 26999604 PMC4811112

[B26] Lin F. Worman H. J. (1993). Structural organization of the human gene encoding nuclear lamin A and nuclear lamin C. *J. Biol. Chem.* 268 16321–16326. 8344919

[B27] Lin F. Worman H. J. (1995). Structural organization of the human gene (LMNB1) encoding nuclear lamin B1. *Genomics* 27 230–236. 10.1006/geno.1995.1036 7557986

[B28] Lombardi M. L. Jaalouk D. E. Shanahan C. M. Burke B. Roux K. J. Lammerding J. (2011). The interaction between nesprins and sun proteins at the nuclear envelope is critical for force transmission between the nucleus and cytoskeleton. *J. Biol. Chem.* 286 26743–26753. 10.1074/jbc.M111.233700 21652697 PMC3143636

[B29] Maresca G. Natoli M. Nardella M. Arisi I. Trisciuoglio D. Desideri M. (2012). LMNA knock-down affects differentiation and progression of human neuroblastoma cells. *PLoS One* 7:e45513. 10.1371/journal.pone.0045513 23049808 PMC3458895

[B30] Moss S. F. Krivosheyev V. De Souza A. Chin K. Gaetz H. P. Chaudhary N. (1999). Decreased and aberrant nuclear lamin expression in gastrointestinal tract neoplasms. *Gut* 45 723–729. 10.1136/gut.45.5.723 10517909 PMC1727735

[B31] Ng K. K. Kaye G. (2013). A case of Lamin C gene-mutation with preserved systolic function and ventricular dysrrhythmia. *Australas. Med. J.* 6 75–78. 10.4066/AMJ.2013.1546 23483212 PMC3593523

[B32] Osmanagic-Myers S. Dechat T. Foisner R. (2015). Lamins at the crossroads of mechanosignaling. *Genes Dev.* 29 225–237. 10.1101/gad.255968.114 25644599 PMC4318140

[B33] Ostlund C. Bonne G. Schwartz K. Worman H. J. (2001). Properties of lamin A mutants found in Emery-Dreifuss muscular dystrophy, cardiomyopathy and Dunnigan-type partial lipodystrophy. *J. Cell Sci.* 114 4435–4445. 11792809 10.1242/jcs.114.24.4435

[B34] Pajerowski J. D. Dahl K. N. Zhong F. L. Sammak P. J. Discher D. E. (2007). Physical plasticity of the nucleus in stem cell differentiation. *Proc. Natl. Acad. Sci. U.S.A.* 104 15619–15624. 10.1073/pnas.0702576104 17893336 PMC2000408

[B35] Patni N. Xing C. Agarwal A. K. Garg A. (2017). Juvenile-onset generalized lipodystrophy due to a novel heterozygous missense LMNA mutation affecting lamin C. *Am. J. Med. Genet. A* 173 2517–2521. 10.1002/ajmg.a.38341 28686329 PMC5593256

[B36] Pugh G. E. Coates P. J. Lane E. B. Raymond Y. Quinlan R. A. (1997). Distinct nuclear assembly pathways for lamins A and C lead to their increase during quiescence in Swiss 3T3 cells. *J. Cell Sci.* 110(Pt 19) 2483–2493. 9410886 10.1242/jcs.110.19.2483

[B37] Ribeiro A. J. Khanna P. Sukumar A. Dong C. Dahl K. N. (2014). Nuclear stiffening inhibits migration of invasive melanoma cells. *Cell. Mol. Bioeng.* 7 544–551. 10.1007/s12195-014-0358-3 25544862 PMC4276563

[B38] Scaffidi P. Misteli T. (2005). Reversal of the cellular phenotype in the premature aging disease Hutchinson-Gilford progeria syndrome. *Nat. Med.* 11 440–445. 10.1038/nm1204 15750600 PMC1351119

[B39] Shimi T. Kittisopikul M. Tran J. Goldman A. E. Adam S. A. Zheng Y. (2015). Structural organization of nuclear lamins A, C, B1, and B2 revealed by superresolution microscopy. *Mol. Biol. Cell* 26 4075–4086. 10.1091/mbc.E15-07-0461 26310440 PMC4710238

[B40] Shimi T. Pfleghaar K. Kojima S. Pack C. G. Solovei I. Goldman A. E. (2008). The A- and B-type nuclear lamin networks: microdomains involved in chromatin organization and transcription. *Genes Dev.* 22 3409–3421. 10.1101/gad.1735208 19141474 PMC2607069

[B41] Swift J. Discher D. E. (2014). The nuclear lamina is mechano-responsive to ECM elasticity in mature tissue. *J. Cell Sci.* 127 3005–3015. 10.1242/jcs.149203 24963133 PMC4095853

[B42] Swift J. Ivanovska I. L. Buxboim A. Harada T. Dingal P. C. Pinter J. (2013). Nuclear lamin-A scales with tissue stiffness and enhances matrix-directed differentiation. *Science* 341:1240104. 10.1126/science.1240104 23990565 PMC3976548

[B43] Sylvius N. Hathaway A. Boudreau E. Gupta P. Labib S. Bolongo P. M. (2008). Specific contribution of lamin A and lamin C in the development of laminopathies. *Exp. Cell Res.* 314 2362–2375. 10.1016/j.yexcr.2008.04.017 18538321 PMC3934841

[B44] Tilli C. M. Ramaekers F. C. Broers J. L. Hutchison C. J. Neumann H. A. (2003). Lamin expression in normal human skin, actinic keratosis, squamous cell carcinoma and basal cell carcinoma. *Br. J. Dermatol.* 148 102–109. 10.1046/j.1365-2133.2003.05026.x 12534602

[B45] Vaughan A. Alvarez-Reyes M. Bridger J. M. Broers J. L. Ramaekers F. C. Wehnert M. (2001). Both emerin and lamin C depend on lamin A for localization at the nuclear envelope. *J. Cell Sci.* 114 2577–2590. 11683386 10.1242/jcs.114.14.2577

[B46] Venables R. S. Mclean S. Luny D. Moteleb E. Morley S. Quinlan R. A. (2001). Expression of individual lamins in basal cell carcinomas of the skin. *Br. J. Cancer* 84 512–519. 10.1054/bjoc.2000.1632 11207047 PMC2363768

[B47] Wang L. Xia J. Li J. Hagemann T. L. Jones J. R. Fraenkel E. (2018). Tissue and cellular rigidity and mechanosensitive signaling activation in Alexander disease. *Nat. Commun.* 9:1899. 10.1038/s41467-018-04269-7 29765022 PMC5954157

[B48] Willis N. D. Cox T. R. Rahman-Casans S. F. Smits K. Przyborski S. A. Van Den Brandt P. (2008). Lamin A/C is a risk biomarker in colorectal cancer. *PLoS One* 3:e2988. 10.1371/journal.pone.0002988 18714339 PMC2496895

[B49] Worman H. J. (2012). Nuclear lamins and laminopathies. *J. Pathol.* 226 316–325. 10.1002/path.2999 21953297 PMC6673656

[B50] Young S. G. Jung H. J. Coffinier C. Fong L. G. (2012). Understanding the roles of nuclear A- and B-type lamins in brain development. *J. Biol. Chem.* 287 16103–16110. 10.1074/jbc.R112.354407 22416132 PMC3351360

[B51] Zuo L. Zhao H. Yang R. Wang L. Ma H. Xu X. (2018). Lamin A/C might be involved in the EMT signalling pathway. *Gene* 663 51–64. 10.1016/j.gene.2018.04.040 29665450

